# Survey of Genotype Diversity, Virulence, and Antimicrobial Resistance Genes in Mastitis-Causing *Streptococcus uberis* in Dairy Herds Using Whole-Genome Sequencing

**DOI:** 10.3390/pathogens12121378

**Published:** 2023-11-22

**Authors:** Monika Zouharová, Ján Matiašovic, Jan Gebauer, Katarína Matiašková, Kateřina Nedbalcová

**Affiliations:** Department of Infectious Diseases and Preventive Medicine, Veterinary Research Institute, 62100 Brno, Czech Republic; jan.matiasovic@vri.cz (J.M.); jan.gebauer@vri.cz (J.G.); katarina.matiaskova@vri.cz (K.M.); nedbalcova@vri.cz (K.N.)

**Keywords:** mastitis, intramammary infection, MLST, sequence type, virulence genes, antimicrobial resistance genes, phylogenetic tree

## Abstract

*Streptococcus uberis* is one of the primary causative agents of mastitis, a clinically and economically significant disease that affects dairy cattle worldwide. In this study, we analyzed 140 *S. uberis* strains isolated from mastitis milk samples collected from 74 cow herds in the Czech Republic. We employed whole-genome sequencing to screen for the presence of antimicrobial resistance (AMR) genes and genes encoding virulence factors, and to assess their genetic relationships. Our analysis revealed the presence of 88 different sequence types (STs), with 41% of the isolates assigned to global clonal complexes (GCCs), the majority of which were affiliated with GCC5. The STs identified were distributed across the major phylogenetic branches of all currently known STs. We identified fifty-one putative virulence factor genes, and the majority of isolates carried between 27 and 29 of these genes. A tendency of virulence factors and AMR genes to cluster with specific STs was observed, although such clustering was not evident within GCCs. Principal component analysis did not reveal significant diversity among isolates when grouped by GCC or ST prevalence. The substantial genomic diversity and the wide array of virulence factors found in *S. uberis* strains present a challenge for the implementation of effective anti-mastitis measures.

## 1. Introduction

*Streptococcus uberis* is one of the most important pathogens of the mammary gland. Mastitis caused by this pathogen is widespread throughout the world [1,2,3,4,5]. The occurrence of mastitis negatively affects the economy of farms and animal welfare. Secondarily, it also contributes to the emergence and spread of bacterial resistance, because large amounts of antimicrobials are consumed to suppress them, both for treatment and prevention, in the drying off period [6,7,8].

*S. uberis* is a common environmental pathogen. It survives in stable environments and is also present on the skin of dairy cows. After direct contact with the teat apex, it can invade the teat canal and cause mastitis. Bedding, in particular, is considered a primary source of environmental pathogens. In addition, intramammary infusions, contaminated teat dips, water used for udder preparation before milking, water ponds or mud holes, skin lesions, teat trauma, and flies are considered possible sources of infection [9]. Typical for *S. uberis* is transient intramammary infection (IMI) (duration up to 1 month) [10], occurring as subclinical or clinical mastitis with varying severity of inflammation. The results of some molecular studies also indicate contagious transmission in some *S. uberis* strains that can spread directly from cow to cow, particularly during the milking process. Contagious *S. uberis* strains may appear as transient IMI, as well as persistent IMI (duration more than 1 month), and may progress to a chronic infection (duration more than 2 months) [10,11,12]. Some studies support the existence of specific persistent strains adapted to the mammary gland, while other studies rather suggest reinfections with prevalent environmental strains [3,13,14,15]. The varying severity of mastitis caused by *S. uberis* is thought to be due to host immune defenses of the udder, but it also depends on the characteristics of the attacking strain, its virulence, and its ability to survive and reproduce in the environment and in the mammary gland [11,12].

Multilocus sequence typing (MLST) is one of the DNA-based techniques that provide insights into the population diversity of *S. uberis* [13,16]. The advantage of this method is a very comprehensive and constantly expanding database (https://pubmlst.org/organisms/streptococcus-uberis; accessed on 10 October 2023), where it is possible to compare types of strains from all over the world [3]. The disadvantage is its limited discriminative power, as it evaluates the variation in the sequences of only seven house-keeping genes, while whole-genome sequencing (WGS) technology allows for the comparison of the whole genome of *S. uberis*. The WGS method is thus more accurate to determine the levels of genetic variability between strains [4,17] and reveal the genetic relatedness of the strains.

WGS enables the complete characterization of strains, revealing a large variety of virulence factors and metabolic capabilities that promote bacterial survival in different environments, including the presence of antimicrobials. The great diversity of virulence factor profiles in *S. uberis* strains [4] indicates the different abilities of the strains to penetrate the mammary gland and resist its defense mechanisms [17]. Studies show that a wide range of *S. uberis* genetic variants can be detected within a single herd [14].

The high genetic diversity, giving individual strains a different spectrum of abilities, may be the reason why anti-mastitis measures often fail and cause problems in the development of an effective vaccine. Monitoring the diversity and other traits of *S. uberis* strains is thus essential for developing strategies to control mastitis caused by this important pathogen. Therefore, the main objective of the present study was to characterize *S. uberis* isolates from clinical and subclinical mastitis in terms of the presence of virulence factors and antimicrobial resistance (AMR) genes, and to assess the molecular diversity using MLST and reveal their genetic relatedness through core genome analysis.

## 2. Materials and Methods

### 2.1. Isolates

Between April 2019 and March 2023, 667 *S. uberis* isolates from 215 farms located in the Czech Republic, were obtained from cases of clinical and subclinical mastitis. Isolates were confirmed via matrix-assisted laser desorption ionization–time of flight mass spectrometry (MALDI-TOF MS) (Bruker Daltonics GmbH, Bremen, Germany) and verified through the detection of the *S. uberis*-specific 16S rRNA gene using polymerase chain reaction (PCR) [18]. Out of these isolates, a random selection of 140 isolates from 74 cow herds underwent whole-genome sequencing.

### 2.2. Whole-Genome Sequencing

#### 2.2.1. DNA Isolation, Sequencing, and Data Processing

Genomic DNA from the 140 selected isolates was isolated using the Qiagen DNeasy Blood and Tissue Kit (QIAGEN GmbH, Hilden, Germany) from bacterial colonies grown on blood agar plates. The Nextera XT DNA Library Preparation Kit (Illumina, Inc., San Diego, CA, USA) was used to prepare a sequencing library. Sequencing was performed as paired-end 2 × 150 bp reads using a NextSeq 500 (Illumina, Inc., San Diego, CA, USA). Raw reads were processed using the Tormes 1.3 pipeline [19]. Assembled raw genomes were annotated using prokka [20], and pan- and core-genome analysis was conducted via Roary 3.13.0 [21] using default settings, both within the Tormes pipeline. A cladogram was inferred from the core genome alignment using FastTree software [22] employing the Jukes–Cantor approximate-maximum-likelihood model. The cladogram tree was drawn using Mega [23]. In our case, the cladogram showed the relatedness between closely related isolates better than a phylogram due to the overall high genetic distance among isolates. The raw reads of isolate sequences were deposited to the SRA database under the BioProject accession number PRJNA1043112 (https://www.ncbi.nlm.nih.gov/sra/PRJNA1043112; accessed on 10 October 2023).

#### 2.2.2. Screening for Virulence Factors

The raw genomes were searched for the presence of virulence factors against the SuPVDB database using a local installation of the blastn software [24]. The search was automated using the Suberis_VF_blast.sh script (https://github.com/matiajan/Suberis_vf_blast_script; accessed on 10 October 2023). The cutoff value for the presence of virulence factors was set at a minimum of 75% coverage of the database’s virulence factor genes and a positive-scoring match value (ppos) of at least 90.

#### 2.2.3. Screening for Antimicrobial Resistance Genes

For AMR gene identification, the genome of each isolate was screened against the Resfinder [25], CARD [26], and ARG-ANNOT [27] databases using Abricate [28] within the TORMES 1.3.0 pipeline.

#### 2.2.4. Multilocus Sequence Typing

The initial sequence type assignment was performed using mlst (https://github.com/tseemann/mlst; accessed on 10 October 2023) within the Tormes v1.3 pipeline [19]. The sequences of the new alleles in the MLST scheme genes [16] were submitted to the PubMLST database [29] (https://pubmlst.org/organisms/streptococcus-uberis; accessed on 10 October 2023), where new allele numbers and sequence types (STs) were assigned. Sequence types were not successfully determined for 5 out of 140 isolates. The online version of Phyloviz 2.0 [30] (https://online.phyloviz.net/index; accessed on 10 October 2023) was used to infer the relatedness of STs identified among isolates collected for this study with all other STs assigned in the PubMLST database up to 10 October 2023.

#### 2.2.5. Principal Component Analysis and Heatmap

Two variables—isolate and gene presence/absence—were included in the principal component analysis (PCA), processed using R software and the R package plugins ggplot2 v 3.3.5 and factoextra v.1.0.7. The heatmap was processed using the pheatmap v.1.0.12 package. All packages are available from the CRAN repository (https://CRAN.R-project.org; accessed on 10 October 2023).

## 3. Results

### 3.1. MLST Characterization

MLST analysis revealed 88 different STs in 140 tested *S. uberis* isolates originating from 74 cow herds (see Supplementary S1). Sixty-nine of these STs were considered novel as they did not match any known STs in the *S. uberis* MLST database (https://pubmlst.org/organisms/streptococcus-uberis; accessed on 10 October 2023). New sequences were submitted to the database and assigned new ST numbers.

The most prevalent ST was ST1135, which was detected in 11 isolates from eight farms. Other prevalent STs included ST307, ST1436, ST876, and ST1775. The detected STs in the isolates and their prevalence on farms are summarized in Table 1. In 72 cases, a certain ST was detected in only one isolate. Up to seven different STs were detected on a single farm.

Sequence types from three global clonal complexes (GCC) were identified. Global clonal complex 5 (GCC5) represented the largest group, accounting for 39.3% of the isolates, while one isolate belonged to GCC86 (0.7%), and another to GCC143 (0.7%). The remaining 59.3% of STs were singletons.

**Figure 1:**
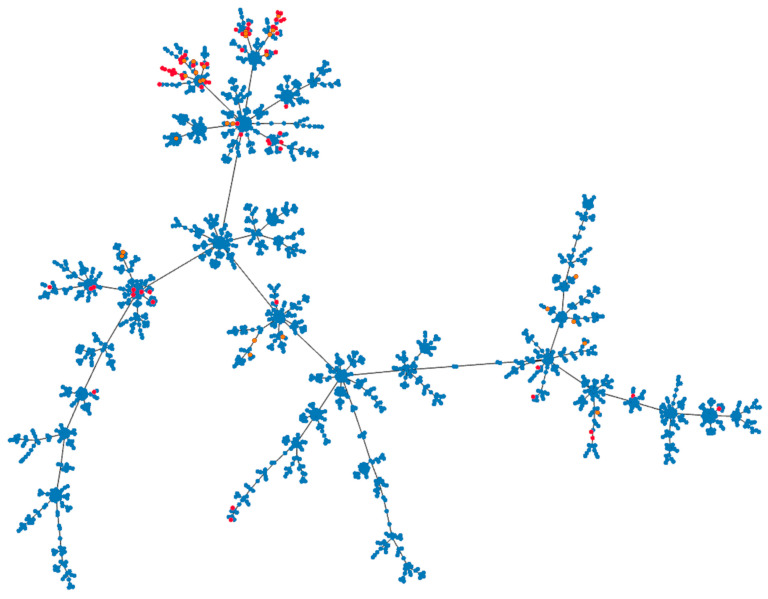
Phyloviz tree based on STs. The red and orange dots represent STs assigned to the isolates analyzed in this study. The red dots indicate the newly assigned STs, while the orange dots represent previously described STs. The blue dots represent all other *S. uberis* STs published in the PubMLST database until 10 October 2023.

The Phyloviz 2.0 tree (Figure 1) showed the presence of the STs identified in this study among all other STs known up to October 10, 2023. The STs identified in this study were distributed across nearly all major branches of the tree, indicating the wide diversity of our *S. uberis* isolates. Additionally, some clonal expansion of newly described STs was observed.

### 3.2. Identification of Virulence Factors

**Figure 2:**
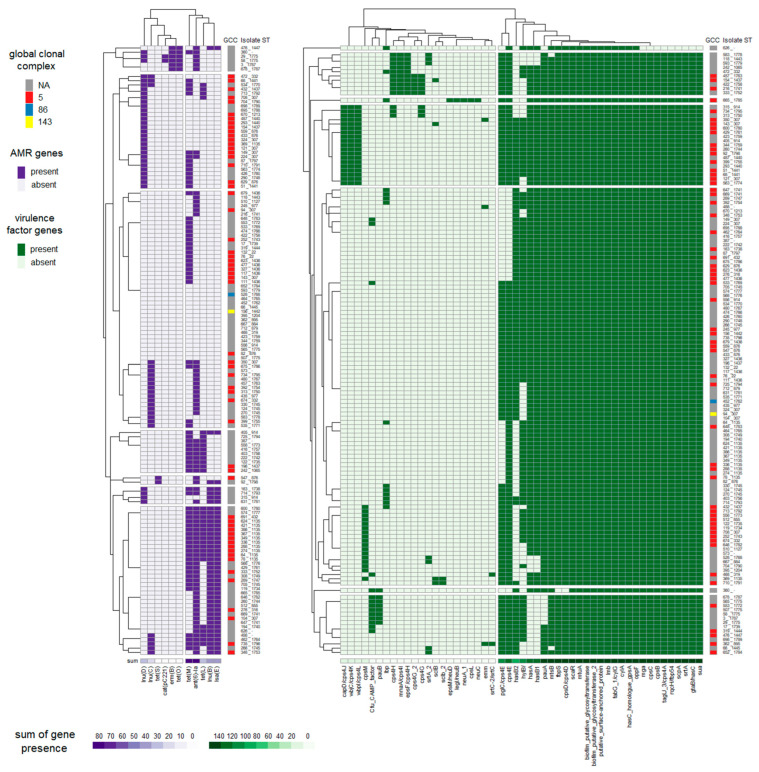
Heat map showing the presence or absence of AMR and virulence genes in isolates. Isolates were clustered according to the presence of virulence or AMR genes. At the bottom of the heat map, the frequency of gene presence is expressed as the sum of occurrence among all isolates.

Fifty-one virulence factor genes from the SuPVDB database were detected in at least one isolate. The number of virulence factor genes per isolate ranged from 18 to 33. Twenty-one of the screened putative virulence factor genes were present in almost every isolate, with only two exceptions. These factors included *biofilm putative glycosyltransferase, biofilm putative glycosyltransferase 2, cylA, fabG 1/cylG, hasC homologue gpsA, lmb, mtuA, oppF, putative surface-anchored protein, scaR, gtaB/hasC, sua, srtA, scpA, rqcH/fbp54, tagU_3/cps4A, cpsB, cpsC, mga, fbpS*, and *cpsD/cps4D* (Figure 2, Supplementary S1). The first ten putative virulence factors were identified in all isolates, while one isolate lacked *gtaB/hasC, sua, srtA, scpA, rqcH/fbp54, tagU_3/cps4A, cpsB, cpsC,* and *mga*, and a second isolate lacked *fbpS* and *cpsD/cps4D*. The majority of isolates harbored between 27 and 29 of the screened virulence factors. The factors *cpsL, neuC, neuA_1, legI/neuB, and epsM/neuD* were found in only one isolate (isolate 665), which belonged to ST1785, a novel ST.

### 3.3. Occurrence of Antimicrobial Resistance Genes

Eighty-seven percent of isolates carried at least one AMR gene. Most of the identified genes were associated with resistance to streptomycin (*ant(6)-Ia* in 54.3% of isolates), lincomycin, and clindamycin (primarily due to the genes *lnu(B)*, *lnu(D)*, and *lsa(E)*), and tetracycline (predominantly due to the *tet(M)* gene). A summary of the occurrence of AMR genes is presented in Table 2.

### 3.4. Association of STs with Virulence and AMR Genes

**Figure 3:**
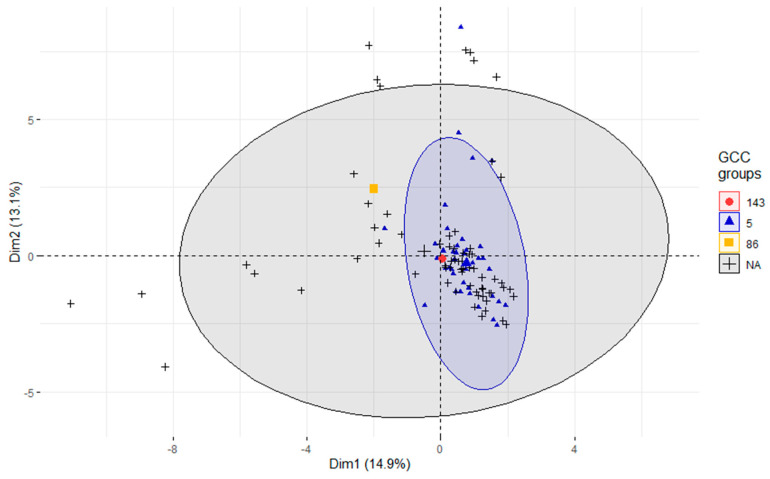
Principal component analysis—grouped by global clonal complexes. PCA grouped according to GCC describes 28% of the total variability expressed by the first two principal components (dimensions). The ellipses represent the 95% confidence interval of all isolates within a group. Two GCC groups are represented by only a single isolate; therefore, confidence ellipses are not available for them.

Principal component analysis was employed to analyze the diversity of isolates grouped by the GCC (refer to Figure 3). Although GCC86 and GCC143 were each identified in only one isolate, GCC5 was identified in 55 samples, while 83 isolates were not assigned to known GCCs. The analysis reveals that isolates from all four groups overlap. As expected, the GCC5 cluster was more compact than the cluster of isolates not assigned to known GCCs. This suggests greater heterogeneity among the latter isolates. From our strain collection, two isolates (626 and 665) were highly divergent from all others (as also seen from the clustering in Figure 2). Therefore, they had to be excluded from the PCA analysis because their inclusion would bias the evaluation of the diversity of the other strains among each other. However, the PCA analysis, including all our isolates, including the outliers, is presented in Supplementary S2.

**Figure 4:**
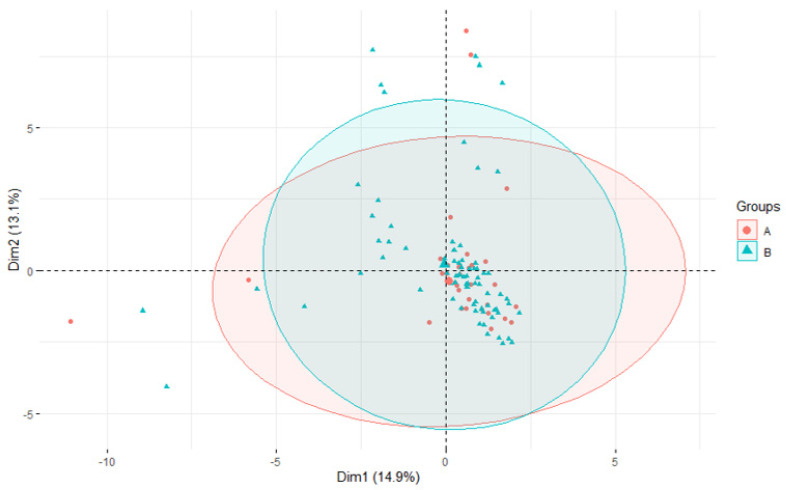
Principal component analysis—grouped by prevalence of STs on farms. PCA grouped according to the prevalence of STs on the farms. The isolates were categorized based on the frequency of ST occurrence into two groups: Group A (highlighted in red) represents the more prevalent STs found on three or more farms, whereas Group B (highlighted in green) includes isolates that occurred on only one or two farms. The ellipses represent the 95% confidence interval of all isolates within a group.

Next, we tested whether the frequently identified STs are associated with a particular set of virulence or AMR genes (Figure 4) that would somehow favor them. The isolates were grouped into two categories: STs present on three or more farms and isolates in which the ST was present on only one or two farms. Once again, both groups overlap, and there is no significant difference between them. Similarly to the previous PCA, the two divergent isolates had to be excluded. The analysis, including all the isolates, outliers included, is presented in Supplementary S3.

**Figure 5:**
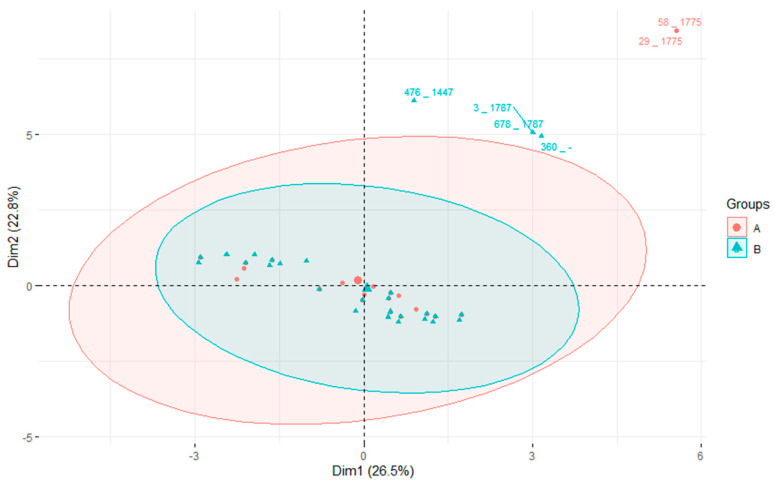
Principal component analysis based on AMR genes and occurrence of STs on farms. In this graph, the isolates are categorized based on the frequency of ST occurrence on the farms. Group A (marked in red) represents the more prevalent STs found on three or more farms, while Group B (marked in green) includes isolates that occurred on only one or two farms. The ellipses represent the 95% confidence interval of all isolates within a group.

**Figure 6:**
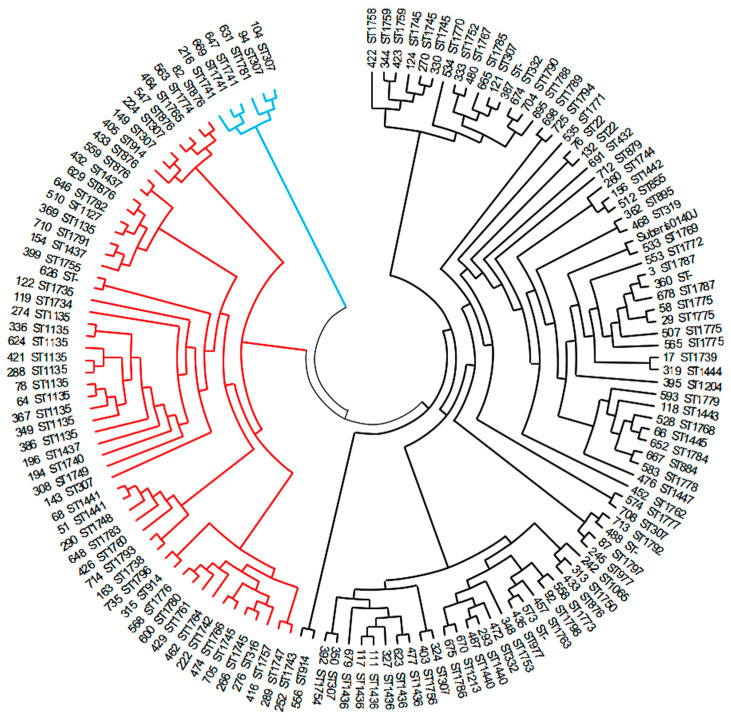
Evolutionary tree based on core genome alignment. The three primary clusters, which shared a common ancestor, are differentiated using color-coded branches (blue, red, black). The cladogram was preferred over the phylogenetic tree due to its superior resolution for closely related isolates.

The heat map in Figure 2 displays the distribution of virulence and AMR genes in all sequenced isolates. The isolates and genes are re-ordered according to the hierarchical clustering, indicating their resemblance. Isolate 626 exhibits a deletion of nine virulence genes, while isolate 665 carries five virulence genes not present in other isolates. Isolate 665 was assigned to ST1785, and this ST was unique to this particular isolate. In line with the PCA analysis, there is no evidence of an association between GCCs and the presence of a specific set of virulence or AMR genes. However, when performing PCA on AMR genes to compare frequent and less frequent STs, it reveals the existence of two small groups of isolates that are distinct from the main cluster. One group consists of four isolates (3, 360, 476, 678), characterized by the presence of tetO and ermB genes, while the other group comprises two isolates (29, 58), identified by the presence of cat(pC221) (Figure 5). Closely related isolates assigned to the same ST tend to share a similar set of virulence and AMR genes (for example, ST1135) (Figure 2). However, this is not an absolute rule. Isolates within ST307, ST914, and ST1745 were found in at least two different branches of the phylogenetic tree constructed based on core genome alignments (Figure 6), indicating heterogeneity in terms of the presence of virulence and AMR genes. 

### 3.5. Phylogeny

Out of the total 5372 genes identified in the draft genomes of all the investigated isolates, pangenome analysis identified 1331 core genes (i.e., genes shared by at least 99% of strains), 170 soft core genes (genes shared by 95–99% of isolates), and 614 shell genes (genes shared by 15–95% of isolates), while 3257 genes were present in less than 15% of isolates.

The core genome comparison revealed three distinct clusters of isolates (see Figure 6). One cluster comprises only six isolates (represented by blue branches), while the other two clusters consist of 54 isolates (red branches) and 80 isolates (black branches), respectively. These two large clusters were further subdivided into numerous subclusters (see Figure 5). Some STs were exclusively present in one cluster (ST1741 and ST1781 in the smallest cluster; ST316, ST876, ST1127, ST1135, ST1437, ST1441, ST1734, ST1735, ST1738, ST1740, ST1742, ST1747, ST1748, ST1749, ST1755, ST1757, ST1760, ST1761, ST1764, ST1765, ST1766, ST1774, ST1176, ST1780, ST1782, ST1783, ST1791, ST1793, and ST1796 in the medium cluster; numerous STs in the largest cluster). Interestingly, ST307 was shared among isolates in all three clusters, while ST914 and ST1745 were present in both large clusters.

## 4. Discussion

Knowledge of genetic relationships and of the presence of virulence factors and AMR genes is important in determining the population of a pathogen occurring in a certain area. The presented results represent a comprehensive study on the characterization of *S. uberis* isolated from intramammary infections. While such information is prevalent in many regions worldwide, it remains largely unavailable in Central and Eastern Europe. To contribute these data to the global understanding of the *S. uberis* population, we conducted an analysis using one hundred and forty *S. uberis* isolates obtained from 74 cow herds situated in the Czech Republic. The isolates underwent genetic characterization, screening for the presence of AMR genes and genes encoding various virulence factors, and their genetic relatedness was assessed through core genome comparison.

While various approaches, such as PFGE [31] or RAPD [32], can be used for typing *S. uberis* isolates, MLST [16] is currently the standard for *S. uberis* typing. As of the end of October 2023, more than 1800 different STs were identified (https://pubmlst.org/organisms/streptococcus-uberis, accessed on 27 October 2023).

In our study, 88 different STs were identified, with the vast majority being isolated only once. Seventy-eight percent of the identified STs were newly described. This diversity of STs aligns with previous studies on mastitis-associated *S. uberis* isolates [2,4], where some studies even found each isolated strain belonging to a different ST [4]. Other studies have reported a significant number of new STs not yet described in the database [3,4]. Only a few STs were shared between two or more countries, with a few STs being shared between countries on different continents, indicating the extensive heterogeneity of *S. uberis* isolates worldwide [33]. The phylogenetic tree encompassing all STs known until October 2023 revealed the presence of our isolates in most major branches of the tree, as well as the clonal expansion of newly identified STs. From these findings, we deduce that some of the *S. uberis* isolates examined in this study belong to a widely distributed global *S. uberis* population, while the remaining isolates may have originated locally. This hypothesis is supported by the introduction of the Holstein breed imported from Western Europe (Danmark, Nedherland, Germany) into Czech herds. The percentage of Holstein cows in Czech herds increased from 0.58% in the year 1970 to 60.8% in the year 2022. While the main import was insemination doses, cows were also brought in. Since the imported cows were not tested for *S. uberis*, they represent a potential source of *S. uberis* strains originating from Western Europe [34,35].

Our exploration of *S. uberis* genotypes also revealed significant heterogeneity of isolates, not only between different locations in the Czech Republic, but also within the same herd, where up to seven STs were identified within a single herd. This is in accordance with a previously published epidemiological study identifying the presence of up to 20 different STs in one herd [36]. During our survey of mastitis-associated *S. uberis* isolates, most STs were identified only on one farm, but several STs occurred more frequently, suggesting that specific strains are more likely to cause intramammary infections than others. In addition, the first two most prevalent STs, namely, ST 1135 and ST 307 (detected on eight and six farms, respectively), were identified previously in other parts of the world (Italy, Croatia) according to the PubMLST database. This suggests that some clones spread more easily than others. Davies et al. also identified nine STs that were significantly more prevalent than the others and were responsible for 38% of clinical mastitis cases in their study, as well as 63% of persistent infections [36].

Our results offered more compact and comparable information when STs were grouped into global clonal complexes, as only three GCCs, specifically, GCC5, GCC86, and GCC143, were regularly determined in studies on clinical and subclinical mastitis. In our study, 41% of isolates were assigned to GCCs, with almost all of them belonging to GCC5. These results support the claim that GCC5 is the major lineage of mastitis-associated *S. uberis* in Europe (e.g., GCC5 occurrence in Switzerland was 30.7%; in the UK, it was 42.5%; in our study, it was 39%) [2,36]. The two other described clonal complexes, GCC86 and GCC143, were found at a minimal occurrence rate of 0.7% each. This reinforces prior findings that the last two mentioned clones are less common in Europe when compared to their prevalence in Australia, New Zealand, or China [4,13,37].

Some studies have also noted an association between the pathogenicity of the strains and the GCC complex, with lower pathogenicity observed in GCC86 compared to GCC5 and GCC143. Strains of STs belonging to GCC86 were mainly isolated from cows with low somatic cell counts [1], and this was associated with the absence of the *hasA* gene, and thus, the absence of capsule formation (75–77.8% of the GCC86 isolates lacked the *hasA* gene) [1,13]. Our isolate, which belonged to GCC86 (ST1786), was also *hasA*-negative. However, it has also been proven that a hyaluronic acid capsule is not required for the development of clinical mastitis in experimental infections [38]. In our study, several strains isolated from acute mastitis were both *hasA*- and *hasB*-negative. Moreover, in a recent study from Australia, GCC86 was isolated from clinical mastitis as frequently as GCC143. In our collection of mastitis-causing strains, only one isolate belonged to GCC86, which may be attributed to the geographic spread of genetic lineages rather than lower pathogenicity.

This study identified a limited number of antibiotic resistance genes, detecting only 11 AMR genes, which aligns with certain previous surveys of dairy herds [4]. However, it deviates from other studies that found a broad and diverse array of antibiotic resistance genes [3,5]. The most commonly identified genes were *tet(M)*, responsible for tetracycline resistance, with a prevalence also reported in other studies [3,4,5], and *ant(6)*, responsible for streptomycin resistance. The presence of the *ant(6)* gene in our collection of isolates was relatively high (54%). In other studies, aminoglycoside resistance was predominantly encoded by genes such as *aph*, *S12p*, and *gidB* [3,39]. Our mastitis isolates of *S. uberis* also showed a high occurrence of genes encoding resistance to lincomycin and clindamycin. However, the representation of individual genes (*lnu(B)*, *lnu(D)*, *lnu(C)*, *lsa(E)*, and *lin(B)*) varied considerably in studies from different locations [3,39]. While gene content may not always correlate with phenotypic results [40,41], possibly due to the presence of silent genes and a lack of gene expression, monitoring AMR genes is crucial for estimating the antimicrobial resistance potential of a bacterial population [40], because silent genes may be activated through mutations or recombination, or when transferred to a new host via horizontal gene transfer. A significant positive outcome of this study is that none of the isolates harbored genes encoding resistance to penicillins, the recommended first-line substance for *S. uberis* intramammary infection treatment, while in some other countries, genes encoding resistance to beta-lactams were often identified (*bl2b*, *tem*, *blaZ*) [5,39].

As the Virulence Factor Database (VFDB; http://www.mgc.ac.cn/VFs/; accessed on 10 October 2023) does not contain *S. uberis* virulence genes, a custom database named the *S. uberis* Putative Virulence Database (SuPVDB), constructed by Vezina et al. [4], was utilized. This database consisted of 53 sequences of virulence factor genes, selected based on experimental studies demonstrating their association with virulence or the identification of homologues from the VFDB. The founders of the SuPVDB database outlined the role of all these virulence factors in their publication [4]. In all isolates, a consistent set of ten virulence genes was identified, suggesting that these virulence factors are adequate for mammary gland colonization. However, it cannot be concluded that this specific set of virulence factors is necessary for pathogenicity, as other virulence elements could potentially compensate for the absence of these genes. Similar scenarios have been observed in *Streptococcus suis* [42]. Furthermore, the existence of an as yet undiscovered essential virulence factor gene crucial for pathogenicity cannot be ruled out. When examining the presence of virulence factor genes or AMR genes in the genome of more prevalent isolates, no predominant gene or cluster of genes was noted over other less prevalent STs. In the PCA, both populations clearly overlapped. However, the PCA revealed the existence of two small groups of isolates carrying distinct AMR genes, clearly separated from the main group of isolates. Both prevalent and less prevalent STs were present in these small populations.

There was a tendency for virulence factors to cluster by certain STs, as well as for AMR genes to cluster with particular STs. For example, ten out of eleven ST1135 isolates harbored the same cluster of AMR genes and nearly the same cluster of virulence factor genes. Similarly, in the core genome tree, these ten ST1135 isolates clustered together, suggesting that they are genetically closely related. The one ST1135 isolate with a different set of AMR and virulence genes clustered in a different branch of the cladogram, indicating its genetic distance from all other ST1135 isolates. This suggests that genetic relatedness could be linked to the presence of AMR and virulence factor genes and that isolates with the same ST can be present in genetically distinct populations. Other studies have also reported that GCCs did not accurately capture the assumed evolutionary lineages of bovine mastitis-associated *S. uberis* isolates [4]. It is uncertain why the same ST, representing a particular allele combination of seven house-keeping genes, is present in genetically distant isolates. However, this finding is further supported by the presence of ST307 in all three main branches of the cladogram and the presence of ST914 and ST1175 in two different main branches. Possible explanations are positive selection in house-keeping genes or recombination in these genes [43]. The Streptococcus genus is considered to be genetically plastic and prone to recombination, which facilitates rapid core genome evolution [44].

The results of our study suggest that sequence typing is not precise enough to allow for the estimation of the level of pathogenicity of *S. uberis* in the bovine mammary gland. Neither core genome-based phylogenetic grouping nor virulence factor profiles closely correspond with STs or GCC typing. Whole-genome sequencing thus seems to be the method of choice for characterizing isolates in further *S. uberis* epidemiological studies. Our findings indicate that all isolates analyzed in our study contain a sufficient set of virulence factors that enable the invasion of mammary gland tissue, survival in the host environment, evasion of the host’s immune defense mechanisms, and internalization within mammary epithelial cells [1]. 

## Figures and Tables

**Table 1:** Distribution of sequence types in *S. uberis* isolates and on farms.

ST	No of Isolates	No of Farms
1135	11	8
307	9	6
**1436**	6	5
876	5	4
**1775**	4	4
**1745**	5	3
914	3	3
**1437**	3	3
**1741**	3	3
332	2	2
977	2	2
**1440**	2	2
**1759**	2	2
**1787**	2	2
22	2	1
**1441**	2	1
Others *	77	58

* Other STs were each found in only one isolate. Numbers in bold indicates new STs, first described in the Czech Republic.

**Table 2:** Antimicrobial resistance genes identified in 140 *S. uberis* isolates.

AMR Gene	Substance Concerned	No of Isolates	Percentage
*ant(6)-Ia*	Streptomycin	75	53.6
*cat(pC221)*	Chloramphenicol	2	1.4
*erm(B)*	Erythromycin; lincomycin; clindamycin	6	4.3
*lnu(B)*	Lincomycin; clindamycin	42	30
*lnu(C)*	Lincomycin	24	17.1
*lnu(D)*	Lincomycin	34	24.3
*lsa(E)*	Lincomycin; clindamycin; tiamulin	42	30
*tet(L)*	Doxycycline; tetracycline	32	22.9
*tet(M)*	Doxycycline; tetracycline; minocycline	71	50.7
*tet(O)*	Doxycycline; tetracycline; minocycline	6	4.3
*tet(S)*	Doxycycline; tetracycline; minocycline	2	1.4

## Data Availability

The new allele sequences and sequence types were submitted to the *S. uberis* MLST database (https://pubmlst.org/suberis, accessed on 26 August 2023).

## Supplementary Materials

The following supporting information can be downloaded at: https://www.mdpi.com/article/10.3390/pathogens12121378/s1, Supplementary S1: Isolate data; Supplementary S2: Principal component analysis, grouped by global clonal complex—all isolates; Supplementary S3: Principal component analysis, grouped by prevalence of STs on the farms—all isolates.

## References

[1] Tomita T., Meehan B., Wongkattiya N., Malmo J., Pullinger G., Leigh J., Deighton M. (2008). Identification of *Streptococcus uberis* multilocus sequence types highly associated with mastitis. Appl. Environ. Microbiol..

[2] Käppeli N., Morach M., Zurfluh K., Corti S., Nüesch-Inderbinen M., Stephan R. (2019). Sequence types and antimicrobial resistance profiles of *Streptococcus uberis* isolated from bovine mastitis. Front. Vet. Sci..

[3] Silva N.C.C., Yang Y., Rodrigues M.X., Tomazi T., Bicalho R.C. (2021). Whole-genome sequencing reveals high genetic diversity of *Streptococcus uberis* isolated from cows with mastitis. BMC Vet. Res..

[4] Vezina B., Al-harbi H., Ramay H.R., Soust M., Moore R.J., Olchowy T.W.J., Alawneh J.I. (2021). Sequence characterisation and novel insights into bovine mastitis-associated *Streptococcus uberis* in dairy herds. Sci. Rep..

[5] Zhang T., Niu G., Boonyayatra S., Pichpol D. (2021). Antimicrobial Resistance Profiles and Genes in *Streptococcus uberis* Associated with Bovine Mastitis in Thailand. Front. Vet. Sci..

[6] Halasa T., Huijps K., Østerås O., Hogeveen H. (2007). Economic effects of bovine mastitis and mastitis management: A review. Vet. Q..

[7] Saini V., McClure J.T., Léger D., Dufour S., Sheldon A.G., Scholl D.T., Barkema H.W. (2012). Antimicrobial use on Canadian dairy farms. J. Dairy Sci..

[8] Martins L., Gonçalves J.L., Leite R.F., Tomazi T., Rall V.L.M., Santos M.V. (2021). Association between antimicrobial use and antimicrobial resistance of *Streptococcus uberis* causing clinical mastitis. J. Dairy Sci..

[9] Wente N., Klocke D., Paduch J.H., Zhang Y., Seeth M.T., Zoche-Golob V., Reinecke F., Mohr E., Krömker V. (2019). Associations between *Streptococcus uberis* strains from the animal environment and clinical bovine mastitis cases. J. Dairy Sci..

[10] Pullinger G.D., Coffey T.J., Maiden M.C., Leigh J.A. (2007). Multilocus-sequence typing analysis reveals similar populations of *Streptococcus uberis* are responsible for bovine intramammary infections of short and long duration. Vet. Microbiol..

[11] Srithanasuwan A., Pangprasit N., Suriyasathaporn W. (2022). Comparison of Virulence Patterns Between *Streptococcus uberis* Causing Transient and Persistent Intramammary Infection. Front. Vet. Sci..

[12] Leelahapongsathon K., Schukken Y., Srithanasuwan A., Suriyasathaporn W. (2020). Molecular epidemiology of *Streptococcus uberis* intramamary infections: Persistent and transient patterns of infection in a dairy herd. J. Dairy Sci..

[13] Pullinger G.D., López-Benavides M., Coffey T.J., Williamson J.H., Cursons R.T., Summers E., Lacy-Hulbert J., Maiden M.C., Leigh J.A. (2006). Application of *Streptococcus uberis* multilocus sequence typing: Analysis of the population structure detected among environmental and bovine isolates from New Zealand and the United Kingdom. Appl. Environ. Microbiol..

[14] Phuektes P., Mansell P.D., Dyson R.S., Hooper N.D., Dick J.S., Browning G.F. (2001). Molecular epidemiology of *Streptococcus uberis* isolates from dairy cows with mastitis. J. Clin. Microbiol..

[15] Zouharova M., Nedbalcova K., Kralova N., Slama P., Matiaskova K., Matiasovic J. (2022). Multilocus Sequence Genotype Heterogeneity in *Streptococcus uberis* Isolated from Bovine Mastitis in the Czech Republic. Animals.

[16] Coffey T.J., Pullinger G.D., Urwin R., Jolley K.A., Wilson S.M., Maiden M.C., Leigh J.A. (2006). First Insights into the Evolution of *Streptococcus uberis*: A Multilocus Sequence Typing Scheme That Enables Investigation of Its Population Biology. Appl. Environ. Microbiol..

[17] Ward P.N., Holden M.T., Leigh J.A., Lennard N., Bignell A., Barron A., Clark L., Quail M.A., Woodward J., Barrell B.G. (2009). Evidence for niche adaptation in the genome of the bovine pathogen *Streptococcus uberis*. BMC Genom..

[18] Hassan A.A., Khan I.U., Abdulmawjood A., Lämmler C. (2001). Evaluation of PCR methods for rapid identification and differentiation of *Streptococcus uberis* and *Streptococcus parauberis*. J. Clin. Microbiol..

[19] Quijada N.M., Rodríguez-L’azaro D., Eiros J.M., Hern´andez M. (2019). TORMES: An automated pipeline for whole bacterial genome analysis. Bioinformatics.

[20] Seemann T. (2014). Prokka: Rapid prokaryotic genome annotation. Bioinformatics.

[21] Page A.J., Cummins C.A., Hunt M., Wong V.K., Reuter S., Holden M.T.G., Fookes M., Falush D., Keane J.A., Parkhill J. (2015). Roary: Rapid large-scale prokaryote pan genome analysis. Bioinformatics.

[22] Price M.N., Dehal P.S., Arkin A.P. (2010). FastTree 2—Approximately maximum-likelihood trees for large alignments. PLoS ONE.

[23] Kumar S., Stecher G., Li M., Knyaz C., Tamura K. (2018). MEGA X: Molecular evolutionary genetics analysis across computing platforms. Mol. Biol. Evol..

[24] Camacho C., Coulouris G., Avagyan V., Ma N., Papadopoulos J., Bealer K., Madden T.L. (2009). BLAST+: Architecture and applications. BMC Bioinform..

[25] Zankari E., Hasman H., Cosentino S., Vestergaard M., Rasmussen S., Lund O., Aarestrup F.M., Larsen M.V. (2012). Identification of acquired antimicrobial resistance genes. J. Antimicrob. Chemother..

[26] McArthur A.G., Waglechner N., Nizam F., Yan A., Azad M.A., Baylay A.J., Bhullar K., Canova M.J., De Pascale G., Ejim L. (2013). The comprehensive antibiotic resistance database. Antimicrob. Agents Chemother..

[27] Gupta S.K., Padmanabhan B.R., Diene S.M., Lopez-Rojas R., Kempf M., Landraud L., Rolain J.M. (2014). ARG-ANNOT, a new bioinformatic tool to discover antibiotic resistance genes in bacterial genomes. Antimicrob. Agents Chemother..

[28] Seemann T. ABRicate Github. https://github.com/tseemann/abricate.

[29] Jolley K.A., Bray J.E., Maiden M.C.J. (2018). Open-access bacterial population genomics: BIGSdb software, the PubMLST.org website and their applications. Wellcome Open Res..

[30] Nascimento M., Sousa A., Ramirez M., Francisco A.P., Carriço J.A., Vaz C. (2017). PHYLOViZ 2.0: Providing scalable data integration and visualization for multiple phylogenetic inference methods. Bioinformatics.

[31] Douglas V.L., Fenwick S.G., Pfeiffer D.U., Williamson N.B., Holmes C.W. (2000). Genomic typing of *Streptococcus uberis* isolates from cases of mastitis, in New Zealand dairy cows, using pulsed-field gel electrophoresis. Vet. Microbiol..

[32] Wieliczko R.J., Williamson J.H., Cursons R.T., Lacy-Hulbert S.J., Woolford M.W. (2002). Molecular typing of *Streptococcus uberis* strains isolated from cases of bovine mastitis. J. Dairy Sci..

[33] Rahman A., Bhattacharjee A., Tabassum T., Islam M.A., Hossain M. (2021). Prevalence and population biology of mastitis-causing *Streptococcus uberis* using an MLST based approach. J. Adv. Biotechnol. Exp..

[34] (2005). Šlechtění Holštýnského Skotu.

[35] (2023). Ročenka 2022–1 Část.

[36] Davies P.L., Leigh J.A., Bradley A.J., Archer S.C., Emes R.D., Green M.J. (2016). Molecular epidemiology of *Streptococcus uberis* clinical mastitis in dairy herds: Strain heterogeneity and transmission. J. Clin. Microbiol..

[37] Wang L., Chen W., Zhang L., Zhu Y. (2013). Genetic diversity of *Streptococcus uberis* isolates from dairy cows with subclinical mastitis in Southern Xinjiang Province, China. J. Gen. Appl. Microbiol..

[38] Field T.R., Ward P.N., Pedersen L.H., Leigh J.A. (2003). The hyaluronic acid capsule of *Streptococcus uberis* is not required for the development of infection and clinical mastitis. Infect. Immun..

[39] Vélez J.R., Cameron M., Rodríguez-Lecompte J.C., Xia F., Heider L.C., Saab M., McClure J.T., Sánchez J. (2017). Whole-Genome Sequence Analysis of Antimicrobial Resistance Genes in Streptococcus uberis and Streptococcus dysgalactiae Isolates from Canadian Dairy Herds. Front. Vet. Sci..

[40] Stasiak M., Maćkiw E., Kowalska J., Kucharek K., Postupolski J. (2021). Silent Genes: Antimicrobial Resistance and Antibiotic Production. Pol. J. Microbiol..

[41] Vezina B., Rosa M.N., Canu A., Tola S. (2022). Genomic surveillance reveals antibiotic resistance gene transmission via phage recombinases within sheep mastitis-associated *Streptococcus uberis*. BMC Vet. Res..

[42] Tram G., Jennings M.P., Blackall P.J., Atack J.M. (2021). *Streptococcus suis* pathogenesis-A diverse array of virulence factors for a zoonotic lifestyle. Adv. Microb. Physiol..

[43] Lynch M. (2023). Mutation pressure, drift, and the pace of molecular coevolution. Proc. Natl. Acad. Sci. USA.

[44] Lefébure T., Stanhope M.J. (2007). Evolution of the core and pan-genome of Streptococcus: Positive selection, recombination, and genome composition. Genome Biol..

