# Supplementary figures and images for: Survey of Genotype Diversity, Virulence, and Antimicrobial Resistance Genes in Mastitis-Causing Streptococcus uberis in Dairy Herds Using Whole-Genome Sequencing
Source: Pathogens. 2023 Nov 22;12(12):1378. doi: 10.3390/pathogens12121378 (PMC10745719; doi:10.3390/pathogens12121378)

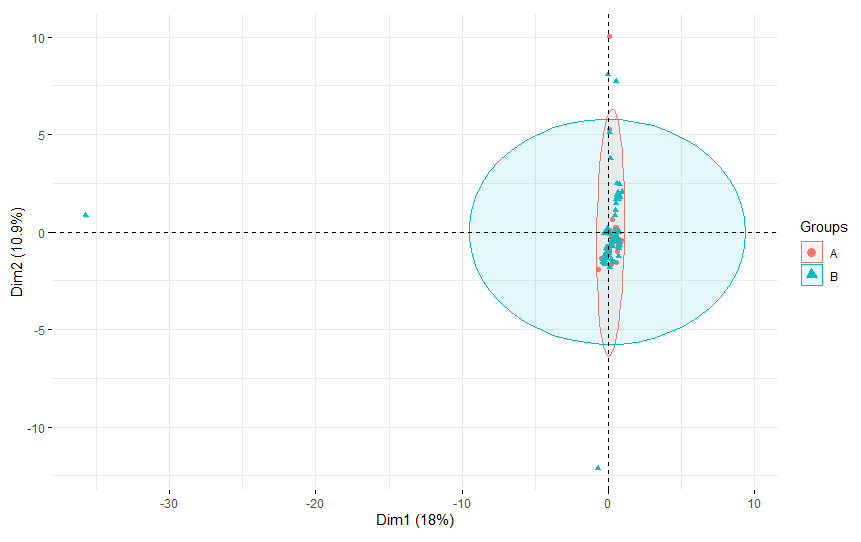

Supplement: Supplementary file 1 [file pathogens-12-01378-s001.zip › Supplementary_Figure_S3.png]

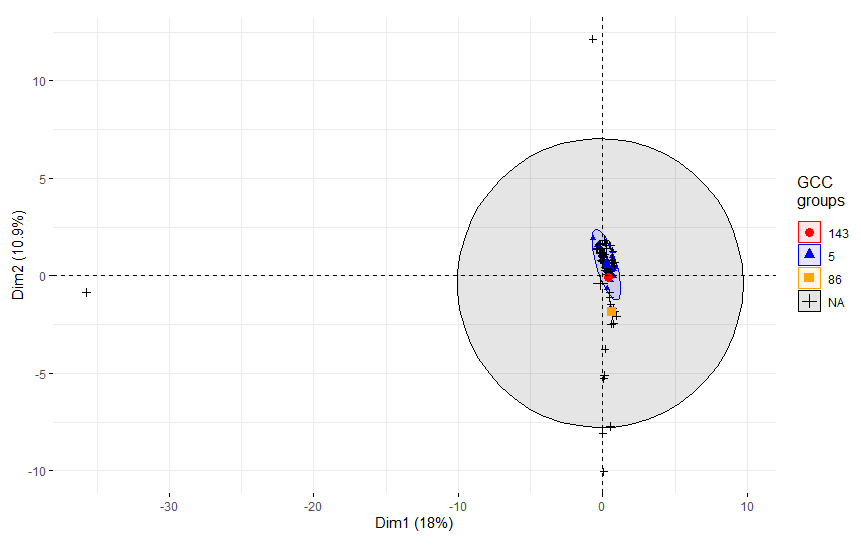

Supplement: Supplementary file 1 [file pathogens-12-01378-s001.zip › Supplementary_Figure_S2.png]
